# White paper on best practices for translational research in neuroendocrine neoplasms

**DOI:** 10.1111/jne.70072

**Published:** 2025-07-28

**Authors:** Jerome Cros, Oriol Casanovas, Justo P. Castaño, Talya Dayton, Alejandro Garcia Alvarez, Benjamin Gibert, Michele Simbolo, Timon Vandamme, Mauro Cives, Ilaria Marinoni, the ENETS Basic and Translational Research Group (BTRG)

**Affiliations:** ^1^ Department of Pathology, Beaujon Hospital (AP‐HP) Université Paris Cité Paris France; ^2^ ProCURE, Oncobell Program Institut d'Investigació Biomèdica de Bellvitge (IDIBELL), Hospitalet de Llobregat Barcelona Spain; ^3^ Maimonides Biomedical Research Institute of Cordoba (IMIBIC) Córdoba Spain; ^4^ Department of Cell Biology, Physiology, and Immunology University of Córdoba Córdoba Spain; ^5^ Reina Sofia University Hospital Córdoba Spain; ^6^ CIBER Fisiopatología de la Obesidad y Nutrición (CIBERobn) Córdoba Spain; ^7^ Tissue Biology and Disease Modelling, European Molecular Biology Laboratory (EMBL) Barcelona Barcelona Spain; ^8^ Gastrointestinal and Endocrine Tumor Unit, Medical Oncology Department Vall d'Hebron University Hospital, Vall d'Hebron Institute of Oncology Barcelona Spain; ^9^ Gastroenterology and Technologies for Health Centre de Cancérologie de Lyon, INSERM U1052‐CNRS UMR5286, Centre Léon Bérard Lyon France; ^10^ Section of Pathology, Department of Diagnostics and Public Health University of Verona Verona Italy; ^11^ Integrated Personalized and Precision Oncology Network (IPPON), Center for Oncological Research (CORE) University of Antwerp and Antwerp University Hospital Wilrijk Belgium; ^12^ NETwerk and Department of Oncology Antwerp University Hospital Edegem Belgium; ^13^ Interdisciplinary Department of Medicine University of Bari “Aldo Moro,” Bari Italy; ^14^ Division of Medical Oncology, A.O.U. Consorziale Policlinico di Bari Bari Italy; ^15^ Institute of Tissue Medicine and Pathology University of Bern Bern Switzerland

**Keywords:** carcinoids, genomics, liquid biopsy, models, transcriptomics

## Abstract

Basic and translational investigations play a crucial role in advancing our understanding of neuroendocrine neoplasms (NENs). In this white paper by the Basic and Translational Research Group of the European Neuroendocrine Tumor Society, we discuss the qualities and drawbacks of current disease models and propose good practices for integrating state‐of‐the‐art technologies including bulk and single‐cell genomics, transcriptomics, and proteomics in contemporary NEN research. We also provide insights on how to properly handle tissue samples (particularly when starting material is limited) and discuss technical hints of relevance when planning liquid biopsy or tumor immunology studies. Future translational studies of NENs will benefit from centralized biologic material biobanking, research design planning in the context of multi‐expertise committees, as well as experimental protocol optimization and sharing across the NEN scientific community.

## 1 INTRODUCTION

Basic and translational investigations are key drivers of advances in oncology. The better understanding of tumor progression and mechanisms of resistance, the discovery of new therapeutic targets and biomarkers are only a few examples. Biological resources are the cornerstone of these studies, and in rare neoplasms such as neuroendocrine neoplasms (NENs) they must be particularly looked after and carefully used. Their quality and the one of tumor models will determine the relevance of the findings and how fast they can be applied to improve clinical care. It is therefore of the utmost importance to have common standardized operating procedures so that resources can be shared or pooled. Similarly, clinical trials in NENs are extremely precious due to the quality of the data that they provide, but they are often lengthy due to the rarity of the disease. Ancillary studies and sampling performed during the trials must be very carefully planned to ensure that they are compatible with current and future state‐of‐the‐art technologies to answer the many questions open in NENs. In this white paper, we will present the most common study models available in thoracic and digestive NENs, their qualities and drawbacks, and propose good practices for tissue sample handling, genomic, transcriptomic, and proteomics analyses, as well as circulating and immune biomarkers evaluation.

## 2 ETHICAL AND LEGAL REQUIREMENTS TO SHARE SAMPLES AND DATA IN BIOMEDICAL RESEARCH

Ethical regulations for sample sharing and analysis are a fast‐moving field with possibly differing inter‐country requirements. This aspect cannot be treated lightly as obtaining the proper documents from all the participating centers may take months, sometimes years. Not completing (or starting) such procedures may impact the perceived feasibility of a project when being reviewed for a grant application. The specifics include multiple types of documents, from informed consents from patients to legal approval from germane ethical committees, which commonly differ substantially depending on the country, and will not be discussed here.

In a multicenter international study using samples, one might consider performing the research in parallel in each center if the technologies can be similar (like federated learning is performed for artificial intelligence). If this is not possible, in addition to the local ethical permit, the researchers will have to obtain the approval for sharing and shipping samples and for the recipient to import them.

Another important aspect to consider is the potential intellectual property that the project may generate or that has already generated in one of the centers. Consortium agreements may take months to years to be signed.

## 3 NEN MODELS

### 3.1 In vitro/ex vivo models

One of the major limitations in the study of NENs is the lack of relevant in vitro models.^1^ In vitro models include cancer cell lines and primary cells freshly isolated from patient samples that can be grown as tumoroids or organoids. In general, cancer cell lines are cells that have adapted to culture conditions and can be expanded in vitro.^1^ Cell lines often lose patients' tumor characteristics but are easy to manipulate using different approaches (transfection, transduction, CRISPR/Cas9, etc.), can be used in a variety of in vitro assays, and can be easily cultured. In general, experiments employing cell lines are relatively cheap and fast. Establishing cell lines from NENs has proven to be difficult.^1^ This is probably due to the low proliferative nature of these tumors and the rarity of the disease. Other in vitro systems include spheroids, patient‐derived tumoroids, or patient‐derived tumor organoids (PDTOs; Table 1) While patient‐derived tumoroids and PDTOs are not easy to manipulate and grow slowly, they retain a high similarity to the tumor of origin.^2^ Tumoroids are organized aggregates of a heterogeneous population of tumor cells and are suitable for short‐term experiments only.^3^ PDTOs, instead, are derived from cell suspensions of patient tumor tissue, cultured in stem‐like media, and can be expanded in vitro indefinitely.^2^ In the case of NENs, they grow very slowly, and experiments using NEN organoids are costly and require a long time. The only low‐grade neuroendocrine tumor (NET) PDTOs that have been successfully established and that have been expanded beyond four passages are pulmonary NET PDTOs.^4^

**TABLE 1:** An overview of 3D models for cancer biology studies.

3D model	Definition
Spheroids	This term is often used to refer to organized aggregates of cells derived from 2D cell lines, grown using a variety of 3D‐cell culture techniques.
Patient‐derived tumoroids	Organized aggregates of a heterogeneous population of cells derived from patient tumor tissue. As cells are not generally sorted, the aggregates contain both tumor cells and some stromal cell populations. The media used for tumoroids often contains FBS and other growth factors. Tumoroids cannot be expanded indefinitely and are suitable for short‐term experiments only.
Patient‐derived tumor organoids	Self‐organizing aggregates of patient‐derived tumor cells that can be expanded in vitro indefinitely. Organoid culture involves growth of cells embedded in a basement membrane gel and a defining feature of this culture system is the use of serum‐free media that is designed to promote the growth of tumor cells. While patient‐derived tumor organoids often contain some stromal cell populations at early passages (P0–P3), later passages contain tumor cells exclusively.

**FIGURE 1:**
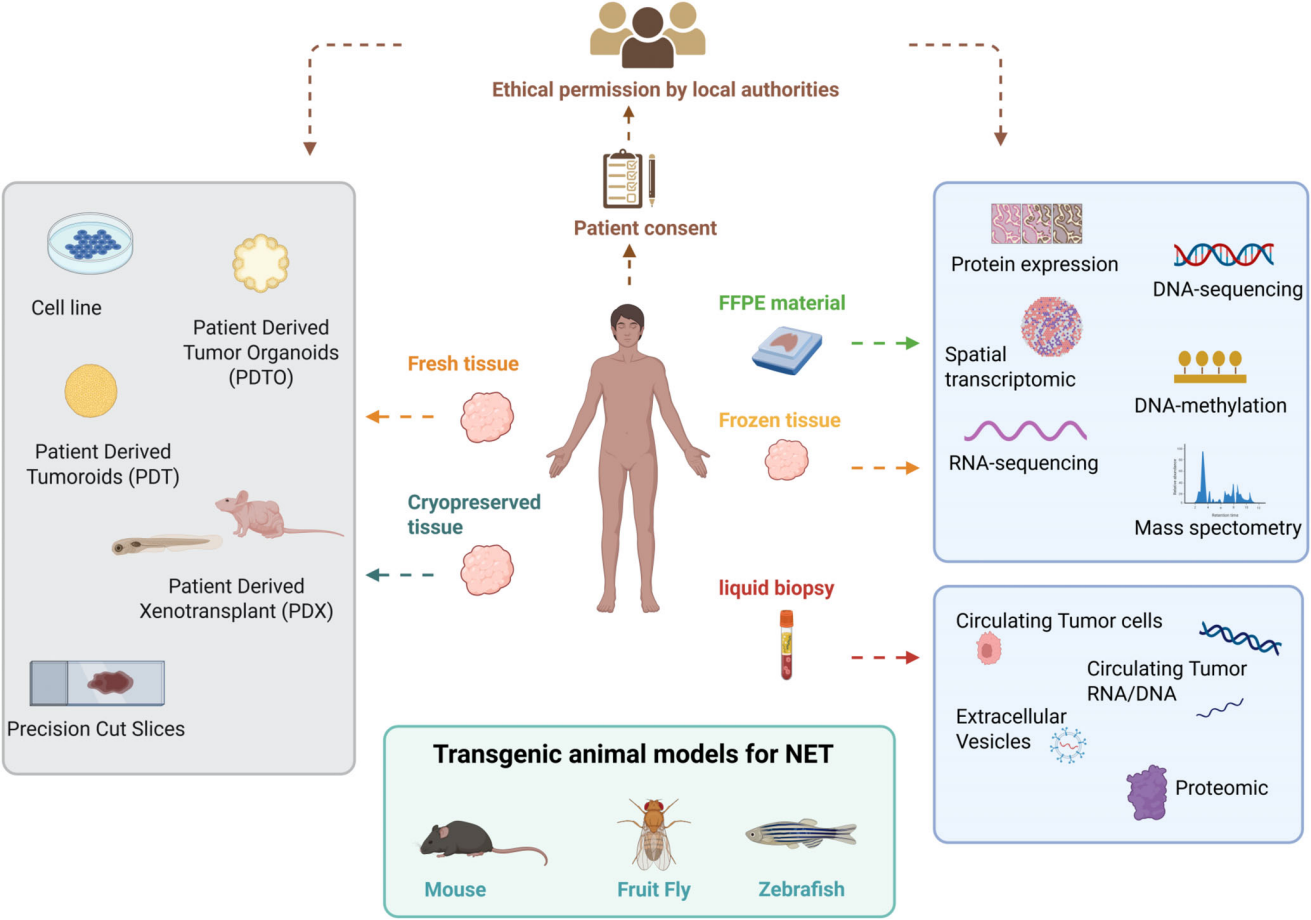
Neuroendocrine neoplasms models and samples for translational studies. Fresh and cryopreserved human tumor samples can be used for generating cell lines, patient‐derived tumor organoids/patient‐derived tumoroids as well as precision‐cut slices. Tumor tissue can also be injected in immunocompromised animal models for generating patient‐derived xenografts. Multiple layers of ‐omic analyses can be performed using formalin‐fixed paraffin‐embedded (FFPE) and frozen tissue. Circulating tumor RNA/DNA, extracellular vesicles, circulating tumor cells as well as circulating immune cells can be isolated and analyzed from blood samples. Genetically engineered animal models can be used to validate biological hypotheses or test the efficacy of therapeutic agents.

When designing the experimental procedure, it is important to consider that each model has some limitations and to choose wisely the most appropriate model according to the scientific question (Table 2). An overview of available NET models is provided in Figure 1.

**TABLE 2:** NET preclinical models: pros, cons, and preferential applications.

Model	Pros	Cons	Preferential applications
Cell lines	Easy to handle and to manipulate	Mainly high grade	Mechanistic studies
PDTs	High efficiency Recapitulate molecular features of patients	Cannot be genetically modified Lack of TME Short‐term use	Drug screening
PDTOs	Recapitulate molecular features of patient Can be genetically manipulated	Mainly high grade Low efficiency and time‐consuming Lack of TME	Drug screening Mechanistic studies
Mouse models	Full TME	Mainly insulinoma Rarely metastases	Studies on angiogenesis Interactions with TME Tumor progression
Xenotransplant (Mouse)	Include part of the TME	Low efficiency in mice Lack of immune system	Drug screening Tumor‐stroma interactions
Xenotransplant (Zebrafish)	Include part of the TME High efficiency	Short‐term use Difference between human and fish biology	Drug screening Cell migration

Abbreviations: NET, neuroendocrine tumor; PDTs, patient‐derived tumoroids; PDTOs, patient‐derived tumor organoids; TME, tumor microenvironment.

#### 3.1.1 Cell lines

A few cell lines derived from pancreatic NEN (PanNEN), small intestine neuroendocrine tumor (SI‐NET) and pulmonary NET are currently available (Table 3). BON1 and QGP1 cells model high‐grade PanNENs rather than well‐differentiated pancreatic neuroendocrine tumors (PanNETs).^5^, ^6^ They are highly proliferative and fundamentally differ from PanNETs in their mutant genetic background.^7^ Nevertheless, they have been important in understanding the biology of PanNENs. Recently, four new PanNET cell lines that closely resemble NET biology (NT3, NT18P, NT18LM, and NT36) have been established.^8^, ^9^ These cell lines have proliferation rates between 10% and 30% and express neuroendocrine markers as well as somatostatin receptors. NT32 and NT38 cells have been isolated from PanNECs. These cells have a proliferation rate of around 50% and display NEC‐typical mutations.^9^

**TABLE 3:** An overview of available NEN cell lines.

Cell line	Characteristics	Source	References
Pancreas			
BON1	Poorly differentiated, *TP53* mutant	PanNEC (lymph node metastasis)	^1^
QGP1	Poorly differentiated, *TP53* mutant	PanNEC	^2^
NT18P	Neuroendocrine marker expression, *MEN1*, *DAXX* mutant	PanNET G3 (primary tumor)	^5^
NT18LM	Neuroendocrine marker expression, *MEN1*, *DAXX* mutant	PanNET G3 (liver metastasis)	^5^
NT36	Neuroendocrine marker expression, *MEN1*, *DAXX* mutant	PanNET G3 (primary tumor)	^5^
NT3	Neuroendocrine marker expression, *MEN1* mutant	PanNET (insulinoma)	^4^
NT32	*BRAF*, *TP53*, and *RB1* mutant	PanNEC	^5^
NT38	*APC* and *ARID1A* mutant	PanNEC	^5^
Small intestine			
GOT1	Loss of chromosome 18	Ileal NET	^7^
CNDT2.5	Neuroendocrine marker expression, serotonin production	Ileal NET (liver metastasis)	^8^
Lung			
TC1, TC2, TC3	Neuroendocrine marker expression	Typical carcinoid	^10^
NCI‐H727	*KRAS* and *TP53* mutant	Typical lung NET	^11^
NCI720	*TP53* mutant, RB1 loss	Atypical lung NET	^11^
NCI‐835	RB1 loss	Typical lung NET	^11^
UMC‐11	RB1 loss	Lung carcinoid	www.atcc.org/

Abbreviations: NEN, neuroendocrine neoplasms; NET, neuroendocrine tumor.

Several SI‐NET cell lines have been reported to date (KRJ‐I, CNDT2.5, GOT1, P‐STS, L‐STS, H‐STS). Unfortunately, one cell line (P‐STS) no longer expresses NET markers, while three other SI‐NET cell lines (KRJ‐I, L‐STS, H‐STS) were found to be derived from transformed lymphoblasts rather than NETs.^10^ GOT1 retains expression of neuroendocrine markers and presents with loss of Shromosome 18, which is common in SI‐NET.^12^ CNDT2.5 was isolated from a liver metastasis of an ileal NET and retains neuroendocrine cell differentiation.^13^ However, the cell line authenticity has been questioned.^14^

Seven pulmonary NET cell lines have been established (NCI‐H727, NCI‐H720, NCI‐H835, UMC‐11, TC1, TC2, TC3). TC1, TC2, and TC3 were derived from typical carcinoids.^15^ These cell lines maintain neuroendocrine marker expression and NET morphology, in addition to showing growth rates consistent with low‐grade NETs. However, following establishment in 2014, these cell lines have not been reported in additional studies. The more commonly used pulmonary NET cell lines are NCI‐H727, NCI‐H720, NCI‐H835, and UMC‐11, but information regarding the derivation of these cell lines is limited (www.atcc.org/). Mutations more often seen in NECs have been reported for NCI‐H727 (activating *KRAS* mutation, loss of function in *TP53*), NCI‐H720 (loss of function in both *RB1* and *TP53*), NCI‐H835 (loss of function in *RB1*), and UMC‐11 (loss of function in *TP53*).

#### 3.1.2 Patient‐derived tumoroids and PDTOs

The culture of patient‐derived NEN tumoroids has been successfully established. After isolation from the tissue, the cells are cultured in 3D. PanNET tumoroids retain neuroendocrine cell differentiation, proliferation rates resembling the tissue of origin, and maintain treatment response similar to the originating tumor.^16^ Tumoroids are typically maintained in culture for 2–3 weeks. In a study using patient‐derived tumoroids of high‐grade NEN, the drug response measured in vitro was shown to correlate with clinical response in patients. In addition, patient‐derived tumoroids highlighted new targets and treatment options for NEC patients.^17^ Similarly, Hien Ear and her team established spheroids from patient‐resected SI‐NETs. SI‐NET spheroids maintain neuroendocrine cell differentiation even after 9 months in culture and are suitable for drug screening.^18^

Patient‐derived tumoroids can be derived either from fresh tumor or cryopreserved tissues. For cryopreservation, tumor tissues can be cut into small pieces (5 mm^3^) and frozen in Recovery Cell Culture Freezing Medium (Thermo Fisher Scientific) in a slow freezing box at −80°C first and then transferred to a nitrogen tank for long storage.^16^ If routinely implemented, biobanking procedures allow gathering large collections of samples ready to use for tumoroid screening. This is particularly valuable in the context of a rare disease such as NENs.

PDTOs have been established for high‐grade NENs originating from a variety of organs, including lung, stomach, liver, duodenum, colon, and pancreas.^19^, ^20^, ^21^, ^22^, ^23^, ^24^ While most of these PDTOs were derived from NECs, three PDTOs were established from G3 NETs: one from a biliary NET, one from a PanNET, and one from a duodenal NET. All the described high‐grade NEN PDTOs were maintained and expanded for more than 6 months, and it is presumed that they can be kept in culture indefinitely. Molecular analysis and expression of neuroendocrine markers confirmed the maintenance of neuroendocrine characteristics and histopathological and molecular phenotypes of the original tumor. Importantly, high‐grade NEN PDTOs could be used for drug sensitivity assays and, where the data was available, the response of the PDTO mirrored the patient response.^20^ In‐depth genomic analyses showed that NEN PDTOs maintain the intratumor heterogeneity of their parental tumor, an important contributor to therapy resistance and tumor evolution.^24^

PDTOs have also been successfully established for low‐grade pulmonary NETs and for a supra‐carcinoid.^4^ When trying to generate NET PDTOs, it is recommended to freeze portions of the culture routinely and as early as possible following the first isolation. Pulmonary NET PDTOs maintain neuroendocrine characteristics and histopathological and molecular phenotypes of their parental tumors. All pulmonary NET PDTOs exhibited slow growth in culture, with an average time to passage of 3 months. The supra‐carcinoid PDTO displayed faster growth in culture and showed a response to targeted therapies that was analogous to the patient response. Low‐grade NET PDTOs are dependent on supplementation of specific growth factors such as epithelial growth factor. PDTOs of GEP NETs have not been established, but it is possible that GEP NETs also have specific growth‐factor requirements that, if identified, could enable the generation of low‐grade GEP‐NET PDTOs.

#### 3.1.3 Precision cut tumor slices

An alternative for short term culture is precision cut tumor slice (PCTS).^24^ Briefly, a tumor core of 1 cm in diameter is taken from the tumor, embedded in low melting agarose, and cut by a vibratome in slices that are around 300 μm thick. These NET slices are then cultured in inserts for several days and can be exposed to multiple drugs. This model has the advantage of retaining the stroma. Slices can be dissociated at the end of the exposure for single‐cell analysis, frozen, or fixed for any downstream application. A personalized immunocompetent PCTS model for NEN liver metastases has been described.^24^ NEN PCTS maintain viability for at least 7 days.

### 3.2 Animal models of NENs

Animal models of NENs have been developed in various species of vertebrate and invertebrate animals, including mice, zebrafish, fruit fly, and dogs. Each of these models has advantages and limitations, and the choice of model depends on the specific research questions being addressed. The development of accurate and relevant animal models is crucial for advancing our understanding of NENs and developing effective treatments.

#### 3.2.1 Genetically engineered mouse models

Mice can be genetically modified to develop NENs by introducing specific mutations that trigger the development of the tumor. A complete list of currently available models can be found in.^25^ Some of these models have been instrumental in advancing our understanding of NETs, in particular in dissecting the heterogeneity of hormone expression^26^ and the role of angiogenesis.^27^ Furthermore, they have helped in the discovery of new targets and the advancement of the therapeutic development of novel drugs to be used in NETs.^28^ These models have the advantage of retaining an efficient immune system. Syngeneic organoids (naïve or modified by CRISPR/Cas9 for instance) can be xenografted in multiple mice for faster experiments.

#### 3.2.2 Xenograft models

Human NEN cells and cell lines can be injected into immunocompromised mice to form xenograft tumors.^29^, ^30^, ^31^ It should be noted that often subcutaneous injection, while convenient, leads to a dense tumor, often necrotic, limited by a thick fibrous capsule with few cancer‐associated fibroblasts and innate immune cells. Orthotopic xenografts, while more cumbersome, may be more relevant. Patient‐derived xenografts have unfortunately a very low success rate.^32^

#### 3.2.3 Zebrafish models

Zebrafish models have emerged as a valuable tool for studying NETs. The transparency of zebrafish embryos allows for easy visualization of tumor growth and progression. Fish embryos injected with human NET cell lines have been used to investigate angiogenesis.^33^ Patient‐derived tumors can be grown in zebrafish embryos to analyze the angiogenic and invasive potential of NETs.^34^, ^35^ Zebrafish embryos and adults can be genetically manipulated to develop NENs.^36^

#### 3.2.4 Fruit fly models

*Drosophila melanogaster* has been utilized as a model organism to study various types of tumors, including NETs (reviewed in^37^). Fruit flies can be genetically modified to express oncogenes or inactivate tumor suppressor genes, leading to the development of neuroendocrine‐like tumors.^38^, ^39^ Such models can be important for studying the therapeutic impingement of genetic insults in NEN initiation and progression.^40^

## 4 NEN TISSUES

Human‐derived tissues and their byproducts are of the utmost importance for NEN research. Standardized sampling protocols and integrative pre‐planned studies are key to sample preservation, allowing its use in multiple studies leading to large “molecularly annotated” cohorts.

### 4.1 Standard operating procedures for sampling

Cold ischemia, namely the time between the removal of the tumor and its processing, is the main cause of tissue/cell degradation leading to organoid culture failure, poor nucleic acid quality, and altered morphology and immunoreactivity. It is therefore important to collect this information so it may be used for sample selection when performing experiments or to interpret discordant data.

**FIGURE 2:**
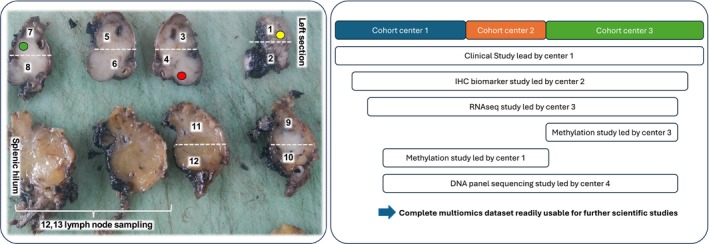
Efficient tissue sampling for research purposes. The left panel shows a distal pancreatectomy that was cut fresh to perform normal (yellow circle) and tumor (red and green circles) tissue sampling that were flash frozen prior to formalin fixation. Areas used for frozen sampling were inked with different colors on the surgical specimen so they can be distinguished, especially when multiple tumor areas are taken. After fixation, slices are placed on a board and photographed. Samples taken are recorded on the photography, and their spatial distribution (white numbers indicating the FFPE block number) is noted. The right panel shows an efficient design of a translational study in which multiple centers merge their cohorts to address different scientific questions. This leads to a well annotated cohort with extensive molecular characterization that can be further enriched by outside teams for additional ‐omic studies.

NENs display an extensive intratumor heterogeneity both at the level of tumor cells and probably in the stromal and immune microenvironment, although this has been less studied. Sampling done on fresh tumors by the pathologist is performed “blindly.” For NENs that are usually well delimited, the sampling is made in the tumor in most cases, but the frozen area might be of a lower grade than the final grade assessed on the complete specimen, hence leading to possible discrepancies when combining molecular and clinicopathological data. It is therefore of the utmost importance to record where the sampling was performed (i.e., which area was used to produce organoids, to make the frozen sampling, etc.). An easy way if multiple samples are taken from the same tumor is to ink each “hole” with a specific color (Figure 2). This will facilitate the sampling of this area on the fixed specimen for proper characterization. In addition, it is important to assess the quality of the frozen sample by performing a histological control by an experienced pathologist before its use for nucleic acid or proteomic extraction (Figure 2).

NEN intratumor heterogeneity and plasticity is poorly understood. NENs can be quite large (primary localization) or have multiple localizations (metastases). It is therefore important that the sampling after formalin fixation for paraffin embedment is extensive to best encompass all the possible tumor components. Taking one sample per cm of the tumor's greater axis is the minimum. Similarly, in the case of multiple metastases, taking at least one sample per lesion >2 cm will ensure correct tumor characterization. Similarly, Ki‐67 assessment on multiple blocks (especially if several morphologies coexist) is recommended to obtain the most accurate tumor grading. For studies devoted to tumor heterogeneity or radio‐pathological correlation studies, a standardized protocol recording the localization of the sampling after formalin fixation will be important. An efficient method is to cut the specimens similarly to CT‐scan images for Whipple resection and perpendicularly to the main duct for left pancreatectomy and to photograph all slices, allowing recording of where all the samples were taken (Figure 2).

### 4.2 Integrative studies for precious samples

Samples such as biopsies or samples derived from a clinical trial are precious. Every time the sample is reused to perform additional studies, a part of it is lost during the process of block adjustment in order to cut the tissue slices that will be deposited on the glass slides to perform immunohistochemistry (IHC), to extract nucleic acids, and so forth. It is therefore tissue‐efficient to plan all the studies in advance through a scientific committee with a molecular pathologist (for samples derived from a clinical trial for instance). This will allow to collect all the requests, prioritize them, and best organize the sample processing in order to minimize tissue loss (example: prepare tissue microarray [TMA] first, so the cores are as long as possible, perform a dual RNA/DNA extraction to limit the number of cut slices, cut slides for IHC by batch in accordance with the project, with 1–2 additional slides in case of IHC failure). Having multiple teams working on the same cohort with different interests also allows to obtain a cohort with multiple levels of molecular annotations that can be further queried by other teams to quickly test a hypothesis. This “mille feuille” approach, while requiring an agreement between the partners for authorship and intellectual property, is the most efficient in the long run (Figure 2).

### 4.3 Tissue microarrays

Whole slide‐based studies are very informative on tumor heterogeneity but can be very time‐consuming or expensive if a large cohort is to be studied. TMAs allow to study multiple tumor fragments on a single slide. Briefly, cores ranging from 600 μm to 2 mm in diameter are taken from the tumors of interest after careful selection by a pathologist. Ideally, at least four cores in different areas are taken per tumor to better model the heterogeneity. Since within the same tumor several blocks may be better preserved than others (exposure to formalin, embedment artifacts), it is recommended that the four cores are taken from at least two blocks if possible. Multiple cores are then placed into an acceptor block that can be cut like any formalin‐fixed paraffin‐embedded (FFPE) block.

## 5 CIRCULATING BIOMARKERS

Liquid biopsies enable minimally invasive detection of tumors, identification of their molecular characteristics as well as patient follow‐up over time and are therefore an attractive alternative for tissue samples. The most investigated source for liquid biopsy is blood, but other biological fluids including urine and saliva can be used as well. These have the advantage of being completely noninvasive and might even be located closer to the primary tumor but contain bacteria, which can potentially dilute the human signal or accelerate its degradation.^41^

### 5.1 Circulating cell‐free nucleic acids

In the acellular fraction of blood obtained after centrifugation, circulating cell‐free nucleic acids including circulating cell‐free DNA (ccfDNA) and RNA (ccfRNA) can be recovered. One of the main challenges for ccfDNA is to avoid contamination with genomic DNA as this dilutes the fraction of ccfDNA and consequently circulating tumor DNA (ctDNA). For this reason, (i) it is recommended to use blood collection tubes with a stabilizing reagent (e.g., Qiagen PAXgene, Streck DNA BCT, Cell‐Free DNA Collection tubes Roche, etc.) and (ii) plasma is preferred over serum.^41^, ^42^ It is widely accepted that plasma is best obtained via a two‐step centrifugation in which a first separation is performed at low speed (800–1600*g*) and then the plasma is centrifuged a second time at high speed (14,000–16,000*g*) for further purification. Storage is at −80°C and multiple freeze–thaw cycles should be avoided.^43^ For ccfRNA, standard K2/K3 ethylenediaminetetraacetic acid (EDTA) tubes appear suboptimal and use of a specialized tube with stabilizer is recommended (e.g., Streck Cell‐Free RNA BCT tube).^44^ Given the difficulties posed by the extraction of ccfRNA, the more stable circulating miRNAs, long noncoding RNAs (lncRNAs) and circular RNAs gained interest. While the tissue‐specific expression of noncoding RNAs makes them ideal circulating biomarkers, inconsistencies have been observed so far when trying to define NET‐specific signatures. No circulating noncoding RNA biomarkers have yet been introduced into routine clinical practice, mostly owing to methodological and standardization problems.^45^ Indeed, standardization and harmonization of pre‐analytical operations are still in their infancy as the optimal matrix, processing method, etc. have not yet been investigated in comparative studies.^41^, ^46^

### 5.2 Circulating tumor cells

Circulating tumor cells (CTCs), also known as “the liquid phase of tumor progression,” can be recovered in the cellular fraction of the blood.^46^ Preservatives specifically aimed at stabilizing CTCs have been developed (e.g., CellSave, Circulating Tumor cell TransFix, sugar‐based cell transport solution, etc.), but their effectiveness is sometimes contradictory.^41^ It is recommended to make a choice depending on the downstream analyses to be performed. It is also recommended not to use the first 5 mL of blood because of possible contamination with epithelial cells of the skin.^47^ After blood collection, enrichment should be performed to increase the CTC concentration to facilitate detection. This enrichment can be based on (i) physical characteristics (e.g., size, electrical charge, etc.), (ii) biological properties (e.g., protein markers on cell membrane), or (iii) a combination of both.^48^ Identification of CTCs can be based on immunological, functional, and/or molecular assays, the latter requiring additional nucleic acid extraction.^41^ Concurrent isolation of ccfDNA and CTCs is possible.^42^

### 5.3 Extracellular vesicles

Extracellular vesicles (EVs) are released by cells throughout the body as part of intercellular communication. Among EVs, exosomes carry specific cargoes such as RNA, miRNAs, proteins, etc. The exosomal RNA is much more stable than the ccfRNA since it is not affected by enzyme activity.^41^ To date, there is no standard protocol for the isolation, processing, and storage of exosomes. Ultracentrifugation methods are usually employed, while other options include polymer‐based systems, immuno‐affinity methods, and filtration systems (based on size), each of which presenting with its own advantages and disadvantages.^41^ Once isolated, the cargo of EVs can undergo downstream analyses. Different extraction methods have been developed, and each of them can influence the cargo itself. Caution should thus be exercised when choosing the extraction method, based on the downstream analyses to be performed.

## 6 TUMOR IMMUNOLOGY

Understanding the intricate interactions between the immune system and NEN cells is of utmost importance for advancing NEN research and paving the way to innovative treatment options for NEN patients. The reliability and validity of tumor immunology studies in NET patients hinge on meticulous sample collection and standardized processing protocols.

### 6.1 Circulating immune cells

Establishing standardized procedures when collecting circulating immune cells is paramount to minimize variability and ensure results reproducibility. First, the choice of the appropriate anticoagulants, such as EDTA, is key to prevent coagulation and preserve the integrity of immune cell populations. Second, acknowledging the influence of circadian rhythms on immune cell composition and activity underscores the importance of consistent timing in sample collection.^49^ Third, the direct or indirect effects of therapeutic agents (not only antitumor agents) on the different immune cell subpopulations should be considered when planning a study on circulating immune cells, and homogeneous patient groups should be evaluated to minimize biases. At this regard, patients should be educated to report any medications to their care providers within the clinical program, and wash‐out periods of specific medications might be discussed in light of the study scopes before collection. Fourth, whether infectious disease marker testing is needed (and within what time frame) should be discussed before collection and should follow institutional guidelines/recommendations. Fifth, proper handling and timely processing of blood samples are imperative to reduce pre‐analytical variability. Since delays in processing can lead to alterations in immune cell populations/activity, prolonged sample storage (>4 h) should be avoided. Cryopreservation of circulating immune cells is possible; a mixture of fetal bovine serum/fetal calf serum and DMSO (with a percentage up to 10% being recommended) can be used to cryopreserve mononuclear blood cells. Commercial products including the CryoStor™ are also available to preserve the quality of cryopreserved T cells.^50^ Cryopreservation of mononuclear blood cells should be routinely implemented in the institutional biobanking procedures to allow gathering a large collective of ready‐to‐use, viable samples. Lysis of red blood cells should be performed before any PBMC analysis, as it improves the quality of flow cytometry and sequencing results.

A variety of technologies to phenotypically and functionally characterize immune cells are currently available. While flow cytometry, multiparameter flow cytometry, fluorescence‐activated cell sorting and mass cytometry are used to phenotypically profile (and possibly isolate) distinct immune cell types, cytokine production assays, tumor cell killing assays, and immune cell proliferation assays are routinely used to evaluate the functional activity of immune cells. A summary of commonly employed markers for immune cell typing and cell differentiation evaluation is provided in Tables 4 and 5, respectively.

**TABLE 4:** Common markers for immune cell phenotyping.

Cell type	Marker
White blood cell	CD45^+^
Granulocytes	CD15^+^
Neutrophils	CD16^+^ CD11b^+^ CD64^+^
Basophils	CD203c^+^
Eosinophils	CCR3^+^ IL5Ra^+^ Siglec‐8^+^
Lymphocytes	CD3^+^
Cytotoxic T cells	CD8^+^
Helper T cells	CD4^+^
Regulatory T cells	CD25^+^ CD127^+^ FoxP3^+^
B cells	CD19^+^
NK cells	CD3^−^ CD56+
NKT cells	CD3+ CD56+
Monocytes	CD14^+^
Dendritic cells	
Classical dendritic cells Plasmacytoid dendritic cells	Lin^−^ HLA‐DR^+^ CD11c^+^ Lin^−^ HLA‐DR^+^ CD123^+^ CD303^+^
Hematopoietic stem cells	CD34^+^

**TABLE 5:** Common markers for T‐ and B‐cell differentiation states.

Cell differentiation state	Marker
T cells	CD3+
Naive	CD45RA^+^ CD45RO^−^ CCR7^+^ CD62L^+^
Central memory	CD45RA^−^ CD45RO^+^ CCR7^+^ CD62L^+^
Effector memory	CD45RA^−^ CD45RO^+^ CCR7^−^ CD62L^−^
Effector	CD45RA^+^ CD45RO^−^ CCR7^−^ CD62L^−^
B cells	CD19^+^
Naive	CD24^+^ CD38^+^
Memory	CD24^+^ CD38^−^
Plasmablasts	CD24^−^ CD38^+^
Regulatory	CD24^+^ CD38^+^ CD1d^+^ CD5^+^

Sequencing the DNA and/or RNA of circulating immune cells is possible by either using traditional bulk approaches or exploiting more recent single‐cell technologies. T cell receptor sequencing can be performed using either DNA or RNA as starting material; the use of RNA allows to better capture the actual TCR expression profile.^51^, ^52^ The inclusion of primers targeting conserved regions across TCR gamma and delta chains (apart canonical alpha and beta chains) can provide insights into unconventional T‐cell subsets.^53^ Adequate sequencing depth is essential for detecting rare T‐cell clones. Established bioinformatic tools (i.e., IMGT/HighV‐QUEST or MiXCR for bulk TCR sequencing; Cellranger‐CDJ, TraCeR or V'DJer for single‐cell TCR sequencing) can be utilized for TCR sequence annotation.^54^, ^55^ Normalization of TCR abundance data is needed to enable meaningful comparisons of clonotype frequencies. As the TCR repertoire is highly dynamic over time, longitudinal TCR sequencing experiments can provide insights into the dynamics of clonal expansion, contraction, and persistence. Cross‐validation of findings from TCR sequencing using protein‐level orthogonal techniques including flow cytometry (employing TCR‐specific antibodies or tetramer staining) or functional assays can ensure the biological relevance of identified T‐cell clones and strengthen the robustness of bioinformatic results.

### 6.2 Tissue‐resident immune cells

FFPE, frozen, or fresh tumor samples can all be analyzed to study immune cell infiltration in NENs. IHC, immunofluorescence, and TMAs are routinely employed to assess the presence and type of immune cells in NENs. RNAseq data can be deconvoluted to depict the immune cell composition of the tumor microenvironment.^56^ To improve the validity and reproducibility of studies focusing on tissue‐resident immune cells, samples collected from different regions of the same tumor (including core and periphery) should be assessed. Multiregional sampling can be indeed useful to obtain a comprehensive understanding of immune cell dynamics within distinct tumor subregions.^57^ When using fresh tumors, minimization of ischemic time is crucial to preserve the viability and functionality of immune cells within the tumor microenvironment. If transport of tumor samples between premises/institutions is needed, the use of cold solutions such as phosphate‐buffered saline on ice can slow down cellular metabolism and preserve immune cell integrity. Cryopreservation of tumor samples (small pieces of approximately 5 mm^3^) utilizing appropriate cryoprotective agents (i.e., culture medium with up to 10% DMSO or Recovery Cell Culture Freezing Medium [Thermo Fisher Scientific], etc.) can be also be exploited. The parallel collection of matched tumor samples and blood samples is particularly important to gain insights on both the systemic and local immunity and is recommended for biobanking purposes.

## 7 GENOMIC STUDIES

### 7.1 DNA sequencing

Five aspects are key for the success of DNA sequencing: (a) high quality and quantity of starting material; (b) choice of the most suitable technology for library preparation; (c) choice of next‐generation sequencing (NGS) platform; (d) choice of the right analysis panel; (e) costs and benefits evaluation.

For the first issue, standard operating procedures are required for the transport of samples to avoid errors and delays in tissue processing.^58^ The type of sample (surgical specimen versus biopsy sample) must carefully be considered. In surgical specimen the tumor is usually well represented, while in a biopsy sample there is sometimes only a small amount of tumor tissue. Other factors including cellularity and tumor characteristics can influence DNA or RNA yield. Low‐cellularity lesions, especially small ones, require multiple unstained sections for nucleic acid extraction.^59^ Overall, a tumor surface ≥1 mm^2^ with a neoplastic cellularity of ≥30% can be considered adequate to obtain a sufficient quantity of nucleic acids for NGS analysis.^60^ Decalcified specimens should not be used for NGS analysis, if possible. Formalin fixation and paraffin embedding should be carried out within 1 h from resection/biopsy, and the window of cold ischemia should be the shortest as possible when RNA extraction is planned.^61^

NGS library preparation systems can induce discrepancies in test reading.^62^ Targeted gene panels use hybridization‐based or amplicon‐based sequencing, where the first provides deep, more uniform coverage and consequently higher sensitivity for variant calling, while the second requires shorter preparation time and smaller DNA input amounts.^63^

There are two main types of NGS platforms: Illumina and Ion Torrent systems.^59^ Platforms differ in chemistry, detection methods, individual specifications, as well as specific error profiles^64^, ^65^ New technologies are available to sequence long fragments (i.e., PACbio, Nanopore, etc.).

The choice of an appropriate gene panel is crucial for the downstream analyses. While small NGS panels containing 5–50 genes may identify a limited number of disease‐relevant mutations, their analysis may be performed quickly and at low cost.^66^ Disease‐oriented panels encompassing recurrently mutated genes in a certain NET type may also be customized.^67^ Whole‐exome sequencing (WES) and whole‐genome sequencing (WGS) may provide a comprehensive coverage of mutations and lead to the discovery of new variants, but costs and interpretative burden (particularly for poorly supported variants) must be carefully considered. A direct comparison between WES and four commercial target panels was recently performed to identify the best approach for detecting the greatest number of alterations of pharmacogenetic relevance in cancer. WES was superior to targeted panels, while the comparison between targeted panels demonstrated that the TrueSight Oncology 500 performs slightly better in identifying pharmaceutically actionable genes.^68^ WGS/WES represent the gold standard for TMB determination. However, numerous studies have found high concordance between the TMB derived from massive sequencing and that obtained by using large gene panels (~300 genes, at least 1 megabase).^69^ The exact cost of DNA sequencing depends heavily on the specific environment and laboratory setup. To date, a decrease in costs for a WES has been observed. However, such costs remain higher when compared to targeted panels.^70^

### 7.2 DNA methylation

Epigenetic includes modifications of DNA that affect gene expression and chromatin organization without altering the DNA sequence. The main epigenetic modifications are DNA methylation, which is the addition of a methyl group to a cytosine, typically in CpG islands, and modification of histone tails. DNA methylation changes during cell development and differentiation. Each cell type has a specific DNA methylation profile. For this reason, DNA methylation profiles are extremely powerful in identifying the cell of origin as well as stratifying tumor subtypes.^71^ DNA methylation analysis can be easily performed on formalin‐fixed tissue. One of the gold standard methods for assessing DNA methylation profiles is the Illumina Infinum MethylationEPIC array and bisulfite WGS. New sequencing technologies are emerging, such as that of Nanopore (not FFPE compatible) allowing to assess the methylation profile and other base modifications. Interestingly, DNA methylation profiles can distinguish three subtypes of PanNETs with different cell origins, genetic backgrounds, and prognoses.^71^ In addition, DNA methylation profiles can distinguish PanNET from PanNEC^72^ and NET from pulmonary, pancreatic, and intestinal origins.^73^

## 8 TRANSCRIPTOMIC STUDIES

### 8.1 Bulk RNAseq

Bulk RNAseq is usually fast and cost effective, but averages the expression levels of a given gene in all the cells present in a sample. Removal of ribosomal RNA (rRNA), which typically constitutes more than 90% of the total RNA in a cell, and consequent enrichment in messenger RNA (mRNA) may be achieved through poly(A) selection or rRNA depletion. Typically, poly(A) selection requires high‐quality, minimally degraded mRNA.^74^, ^75^ For many biologically relevant samples (i.e., tissue biopsies, CTCs, etc.), obtaining good mRNA integrity may be challenging.^76^ Poly(A) selection eliminates all transcripts that are not polyadenylated, including miRNAs, small nucleolar RNAs, rRNAs, certain lncRNAs, circular RNAs, and even certain coding RNAs that have no poly‐A tail or a relatively short tail. Several strand‐specific protocols, such as the widely used deoxyuridine triphosphate method, were developed and offer information on the presence of other RNA types as well as the strand where they were originally transcribed.^77^ Ribosome depletion allows the evaluation of lncRNA, and possibly the microbiome, while miRNAs require a dedicated RNAseq process. Although technologies are evolving very rapidly, transcriptome quality is still better from fresh or frozen tissue. Nevertheless, RNAseq on FFPE tissue is now of good quality. For FFPE samples, protocols using 3′ TAG sequencing technology are the cheapest but require at least 50 ng of RNA as input (not achievable in some biopsies). Ribodepletion‐based protocols are highly efficient for FFPE samples, a bit more expansive but with lower RNA input requirement (10 ng).

RNA sequencing can involve single‐end (SE) or paired‐end (PE) reads, where the latter is preferable for de novo transcript discovery or isoform expression analysis^78^, ^79^ and is imperative in studies focused on RNA splicing.^80^ Longer reads also improve the mapping process and transcript identification.^79^ Considering costs, the cheaper option is represented by short SE reads, whereas longer and PE reads are preferable to characterize poorly annotated transcriptomes. Sequencing is generally carried out at 30× to properly cover the transcriptome. Nevertheless, 100× runs can be required to analyze rare alternative splice variants. Deep sequencing improves quantification and identification of transcripts but may also result in the detection of transcriptional noise and off‐target transcripts.^81^ A minimum of 40 million reads per sample is recommended.^75^

RNAseq is subjected to a significant batch effect that can potentially create discrepancies in the bioinformatic analysis. It is therefore recommended to perform RNAseq for the same cohort at the same time and on the same machine. Grouping RNAseq experiments is also cost‐effective.

Sequencing of noncoding RNAs should be carried out using specific approaches.^82^ Small RNAseq is a high‐throughput sequencing technology specifically designed to identify and quantify small RNA molecules (typically 18–40 nucleotides in length). Enrichment of circular RNAs can be achieved by linear RNAs depletion through RNase R treatment. Short‐read platforms should preferably be used for sequencing noncoding RNAs. Rigorous size selection is of utmost importance when performing small RNAseq to exclude degraded RNA. A fast and cost‐effective profiling of known miRNAs can be achieved using microarray. Nevertheless, small RNAseq should be preferred versus microarray when the identification of novel miRNAs and/or higher sensitivity is required.

### 8.2 Single‐cell RNAseq

Single‐cell RNAseq (scRNAseq) identifies the different cell types present in a cell suspension based on gene expression patterns. It can also be used to identify markers specific to each tumor subpopulation (tumor cells, immune cells, endothelial cells, etc.), or to measure differentially expressed genes between different subpopulations. Rare cell populations can be sorted prior to single‐cell analysis,^83^ whereas populations with low mRNA expression may benefit from a preliminary real time‐polymerase chain reaction amplification phase for proper detection.^84^ Tissue preservation and dissociation are crucial technical aspects of scRNAseq. If possible, experiments should be carried out using fresh tumor tissue right out of the operating theater. Protocols have been developed to perform single‐nuclei RNAseq from frozen samples using a prior step of nuclei isolation. It should be noted that some protocols are now adapted to FFPE samples.

### 8.3 Spatial transcriptomics

Spatial transcriptomics is a technology that combines high‐throughput profiling of gene expression with the spatial information of cells in tissues or organs. It enables the identification and mapping of gene expression patterns of individual cells in their native tissue context, offering a more complete understanding of the molecular and cellular organization of tissues. This technology involves positioning tumor tissue sections on slides containing spots with spatial barcode probes capable of capturing mRNA that will indicate their provenance after sequencing. These experiments take place on arrayed slides comprising around 5000 spots of 50–100 μm in diameter, thus providing a resolution of ~5–20 cells per spot. Resolution is posed to increase rapidly, possibly enabling us to map intracellular interactions in the near future. Any intact tissue containing viable mRNA is suitable for spatial transcriptomics.

Among the most established technologies, 10× Visium has an extensive list of optimized tissues covered^85^ and assesses approximately 18,000 genes, with several genes (i.e., transcription factors) inherently less transcribed than others.^86^ A DV200 (% of RNA fragments >200 nucleotides) of ≥50% is recommended. The 10× Xenium technology delivers high‐plex in situ analysis at subcellular resolution with nanometer precision and can assess up to 5000 genes, achieving fast and robust single‐cell spatial insights.^87^ The Nanostring GeoMx is another widely used technology. Here, small areas on the whole slide are chosen by the scientist, and cell populations are physically isolated for bulk RNAseq. This allows to have, for instance, the separate transcriptome of CD8^+^ T cells, tumor cells, and fibroblasts in multiple areas of the tumor. Facing this microbulk approach (around 10–50 cells lysed on a spot), other technologies have emerged using fluorescence in situ RNA hybridization ± barcode amplification. They comprise either targeted approaches such as RNAscope (2–3 transcripts; immunofluorescence can be added) or larger approaches. In the latter, the number of transcripts is still limited (1000 max) and needs to be predefined. This approach covers a larger area than the 10× Visium but is much more expensive.

## 9 PROTEOMIC STUDIES

Proteomics encompasses the examination of the complete array of proteins expressed within a cell, tissue, or organism. It serves as a valuable complement to genomics and transcriptomics since the data derived from the latter offer only indirect assessments of cellular conditions, potentially failing to precisely mirror concurrent protein alterations.^88^ Proteomic analyses can be conducted using various sample sources, such as biologic fluids, frozen tissue, or FFPE samples, with applicability in the latter being limited to certain techniques due to protein cross‐linking. Blood samples intended for protein biomarkers identification should be processed within 2 h of collection to reduce proteolysis and degradation. Plasma sample aliquots obtained through sequential steps of centrifugation should be immediately stored at −80°C. Sample integrity should be assessed before use by measuring the hemolysis index (absorbance 414/578 ratio) and total protein yield batch‐to‐batch consistency (by Bradford assay or bicinchoninic acid assay).

### 9.1 Mass spectrometry

Mass spectrometry (MS)‐based techniques have found extensive use in the identification and quantification of proteins. They are best suited to fresh or frozen samples, but protocols are now available for FFPE samples. Proteins are extracted and purified from tissue or cell lysates by centrifugation and filtration. Then, the protein mixture is typically separated by two‐dimensional gel electrophoresis to reduce sample complexity. Total proteins can be identified by MS analysis of their peptides, which are produced by enzymatic digestion, and the data are interpreted using a proteome database.^89^ The high‐throughput technologies based on this technique enable semi‐quantitative and quantitative analyses.^90^ MS can also be performed in situ (MALDI imaging), allowing to retain the spatial distribution of the proteome. This spatial approach makes the protein identification less easy, and once peptides of interest have been selected, an additional classical protein identification through MS might be necessary.

### 9.2 Protein pathway array

The protein pathway array (PPA) serves as a high‐throughput method for investigating the regulation of protein–protein interactions, pathway–pathway interactions, and biological functions, aiding in determining the placement of newly identified proteins within cellular signaling networks.^91^ This proteomic approach involves immobilizing protein lysate on a solid surface, followed by probing with antibodies. PPAs enable the assessment of the activation state of critical cellular pathways with antibodies targeting both total and phosphorylated proteins.^92^

### 9.3 Multiplex IHC

Multiplex IHC capability has recently expanded. Either frozen tissue or FFPE specimens can be used as sample sources.^93^ Depending on the technology chosen, the whole slide or a smaller area can be imaged. Targeted approaches allow assessing up to seven markers with the opal fluorochromes. High‐throughput technologies can instead stain from 40 to 100 markers in a single experiment. Staining is performed by rounds of two or four markers, and large panels can therefore take 2–3 days to run on two slides, thus posing limitations on the size of the cohort. Knowledge should be shared between multiplex IHC users for good antibodies and experimental conditions to be applied in the NEN field.

## 10 OMICS DATA INTEGRATION

In the omics era, data integration between different technologies remains a hurdle due to the complexity of datasets and technologies used. Often, the analyzed samples are not extracted at the same time and from the same tumor area, thus potentially biasing the analyses. Multi‐Omics Factor Analysis (MOFA) is a factor analysis model that provides a general framework for integrating multi‐omics datasets in an unsupervised manner. MOFA can be described as a generalization of principal component analysis to multi‐omics data. Given multiple data matrices containing measurements of multiple types of omics data on the same or overlapping sample sets, MOFA infers a low‐dimensional representation interpretable in terms of a few latent factors. These learned factors represent the main sources of variation between data modalities and facilitate the identification of cellular states or tumor subgroups. Such an approach has already been used to distinguish molecular subtypes of small bowel and lung NETs.^94^, ^95^ In the future, data integration through artificial intelligence‐driven algorithms might enable the identification of new, unknown, and unforeseen molecular traits/pathways.

## 11 CONCLUSIONS

Many fundamental questions about the pathobiology of NENs remain unanswered. While currently available research models are limited, novel technological developments are arising both in terms of new in vitro models as well as improved approaches to analyze tissues, especially FFPE tissues, forecasting a bright future for NEN research. In this context, translational studies on NENs will greatly benefit from (i) centralized biologic material biobanking (at institutional, national and international level), (ii) careful research design planification with multi‐expertise committees, (iii) knowledge sharing of the best approaches and caveats to address a specific question, (iv) protocol sharing across the NEN scientific community to make data comparable, (v) inter‐team mutualization of sequencing batches to reduce costs and improve data aggregation. Importantly, ENETS Centers of Excellence should actively promote translational research in NENs, fostering the scientific discoveries that will eventually lead to the cure of these malignancies.

## AUTHOR CONTRIBUTIONS

**Jerome Cros:** Conceptualization; writing – original draft; writing – review and editing; supervision. **Oriol Casanovas:** Writing – original draft; writing – review and editing. **Justo P. Castaño:** Writing – original draft; writing – review and editing. **Talya Dayton:** Writing – original draft; writing – review and editing. **Alejandro Garcia Alvarez:** Writing – original draft; writing – review and editing. **Benjamin Gibert:** Writing – original draft; writing – review and editing. **Michele Simbolo:** Writing – original draft; writing – review and editing. **Timon Vandamme:** Writing – original draft; writing – review and editing. **Mauro Cives:** Writing – original draft; conceptualization; writing – review and editing; supervision. **Ilaria Marinoni:** Conceptualization; writing – original draft; writing – review and editing; supervision.

## CONFLICT OF INTEREST STATEMENT

The authors declare no conflicts of interest.

## PEER REVIEW

The peer review history for this article is available at https://www.webofscience.com/api/gateway/wos/peer‐review/10.1111/jne.70072.

## Data Availability

Data sharing is not applicable to this article as no new data were created or analyzed in this study.
